# Metabolites related to purine catabolism and risk of type 2 diabetes incidence; modifying effects of the *TCF7L2*-rs7903146 polymorphism

**DOI:** 10.1038/s41598-019-39441-6

**Published:** 2019-02-27

**Authors:** Christopher Papandreou, Jun Li, Liming Liang, Mònica Bulló, Yan Zheng, Miguel Ruiz-Canela, Edward Yu, Marta Guasch-Ferré, Cristina Razquin, Clary Clish, Dolores Corella, Ramon Estruch, Emilio Ros, Montserrat Fitó, Fernando Arós, Lluís Serra-Majem, Nuria Rosique, Miguel A. Martínez-González, Frank B. Hu, Jordi Salas-Salvadó

**Affiliations:** 1 Human Nutrition Unit, Faculty of Medicine and Health Sciences, Institut d’Investigació Sanitària Pere Virgili, Rovira i Virgili University, Reus, Spain; 2 CIBER Fisiopatología de la Obesidad y Nutrición (CIBERObn), Instituto de Salud Carlos III, Madrid, Spain; 3 Department of Nutrition, Harvard T.H. Chan School of Public Health, Boston, MA USA; 4 Departments of Epidemiology and Statistics, Harvard T.H. Chan School of Public Health, Boston, MA USA; 5 University of Navarra, Department of Preventive Medicine and Public Health, Pamplona, Spain; 6 Broad Institute of MIT and Harvard University, Cambridge, MA USA; 7 Department of Preventive Medicine, University of Valencia, Valencia, Spain; 8 Department of Internal Medicine, Department of Endocrinology and Nutrition Institut d’Investigacions Biomediques August Pi Sunyer (IDI- BAPS), Hospital Clinic, University of Barcelona, Barcelona, Spain; 9 Lipid Clinic, Department of Endocrinology and Nutrition Institut d’Investigacions Biomediques August Pi Sunyer (IDI- BAPS), Hospital Clinic, University of Barcelona, Barcelona, Spain; 10 Cardiovascular and Nutrition Research Group, Institut de Recerca Hospital del Mar, Barcelona, Spain; 11 Department of Cardiology, University Hospital of Alava, Vitoria, Spain; 12 Department of Clinical Sciences, University of Las Palmas de Gran Canaria, Las Palmas, Spain; 13 Channing Division for Network Medicine, Department of Medicine, Brigham and Women’s Hospital and Harvard Medical School, Boston, MA USA

## Abstract

Studies examining associations between purine metabolites and type 2 diabetes (T2D) are limited. We prospectively examined associations between plasma levels of purine metabolites with T2D risk and the modifying effects of transcription factor-7-like-2 (*TCF7L2*) rs7903146 polymorphism on these associations. This is a case-cohort design study within the PREDIMED study, with 251 incident T2D cases and a random sample of 694 participants (641 non-cases and 53 overlapping cases) without T2D at baseline (median follow-up: 3.8 years). Metabolites were semi-quantitatively profiled with LC-MS/MS. Cox regression analysis revealed that high plasma allantoin levels, including allantoin-to-uric acid ratio and high xanthine-to-hypoxanthine ratio were inversely and positively associated with T2D risk, respectively, independently of classical risk factors. Elevated plasma xanthine and inosine levels were associated with a higher T2D risk in homozygous carriers of the *TCF7L2*-rs7903146 T-allele. The potential mechanisms linking the aforementioned purine metabolites and T2D risk must be also further investigated.

## Introduction

Purines are constituent parts of nucleotides and nucleic acids, playing several roles in human physiology, affecting tissue function, cell integrity, and oxidation. In catabolism, their monophosphate forms are converted to inosine and guanosine; purine nucleoside phosphorylase converts them to hypoxanthine and guanine, respectively. Xanthine oxidase (XO), and guanine deaminase, convert them to xanthine. Finally, xanthine is again oxidized by XO to uric acid^1^.

Blood levels of uric acid are well regulated in healthy subjects but when hyperuricemia occurs, its chronicity has been associated with gout, muscular pain, cardiovascular or/and renal disease and type 2 diabetes (T2D)^2^. Concerning other purine metabolites, elevated plasma allantoin concentrations (8-fold higher) were reported in patients with diabetes, compared to healthy controls^3^, indicating an increased oxidative stress in this metabolic condition. In addition, studies in erythrocytes of patients with diabetes^4^ indicated increased adenine and guanine de-phosphorylation, as well as increased adenosine, inosine, guanosine, and hypoxanthine concentrations, together with a higher turnover ratio of nucleotides and hyper-metabolism, when compared to that in healthy subjects.

In regard to the enzymes involved, compared to healthy controls, serum XO activity was found increased in patients with T2D^5^. Similarly, in patients with metabolic syndrome, XO activity was increased and associated with inflammatory or oxidative status markers^6^. In addition, the activity of adenosine deaminase (ADA), an enzyme that converts adenosine into inosine, was found increased significantly in T2D patients, correlating positively with blood glucose levels^7^. Since adenosine increases glucose uptake into cells, its deamination in insulin-sensitive tissues may be considered a homeostatic cell-protecting adaptation^8^.

Studies that examine cross-sectional or/and longitudinal associations among peripheral levels of uric acid and other purine metabolites, diabetes risk factors, and diabetes incidence are limited^9^. Likewise, although the transcription factor-7-like-2 (*TCF7L2*) rs7903146 polymorphism is one of the main genes associated with increased T2D risk, its interactions with purine metabolites overall^10^, or involvement in genetically linking T2D with gout development^11^, remain unknown. Therefore, in the present prospective study, nested in the framework of the PREDIMED trial, we examined associations between plasma levels of purine-catabolism metabolites or relevant ratios (precursor-product) and T2D risk. Furthermore, we examined whether the *TCF7L2*-rs7903146 polymorphism modifies these associations.

## Methods

### Study design and population

This study was nested, as an unstratified case-cohort study, within the PREDIMED study (ISRCTN35739639), a cardiovascular primary-prevention trial conducted in Spanish primary healthcare centers. The methods and design of the PREDIMED trial has been described in detail elsewhere^12^. The present case-cohort study comprises a random selection of 694 non-diabetic participants (approximately 20%) from the eligible subjects of the PREDIMED cohort without T2D at study inception (3,541 participants were free of T2D at baseline) and with available blood samples, together with all incident cases of T2D that occurred during the follow-up with available plasma samples (251 out of the 273 incident cases). Of the 892 participants included in our analyses, 641 were in the sub-cohort (including 53 overlapping cases) and 198 were the rest of the T2D incident cases, giving a total of 251 cases (Supplemental Fig. 1). Of these, 686 participants out of the 892 had available samples after 1-year of follow-up and were included in the 1-year changes analyses (Supplemental Fig. 1). The Research Ethics Committees (RECs) for each of the recruitment centers approved the study protocol, and participants provided written informed consent. All analyses were performed in accordance with the relevant guidelines and regulations. The RECs were: del Hospital Clinic de Barcelona, del Hospital Universitari Sant Joan de Reus, de la universidad de Valencia, de la Universidad de Navarra, de la Facultad de Medicina, de la Universidad de Málaga, de Euskadi, del Complejo Hospitalario Universitario Insular- Materno Infantil (CHUIMI) del Servicio Canario de Salud, Las Palmas de Gran Canaria, del Parc de Salut Mar, de les Balearic Islands.

### Study samples and metabolite profiling

Fasting (for ≥8 hours) plasma EDTA samples (baseline and at the end of 1-year of follow-up) were collected from subjects and stored at −80 °C. In June 2015, pairs of samples for each participant were randomly ordered and shipped on dry ice to the Broad Institute (Boston, Massachusets, USA) for metabolomics assays. Metabolites related to purine catabolism, including uric acid, allantoin, xanthine, hypoxanthine, inosine, adenosine and guanosine, were semi-quantitatively profiled using liquid chromatography-tandem mass spectrometry (LC-MS) on a system comprised of a Shimadzu Nexera X2 U-HPLC (Shimadzu Corp., Marlborough, MA, USA), coupled to a Q Exactive hybrid quadrupoleorbitrap mass spectrometer (Thermo Fisher Scientific)^13^. Metabolite identities were confirmed using authentic reference standards. Raw data were processed using TraceFinder software (Thermo Fisher Scientific) and Progenesis QI (Nonlinear Dynamics; Newcastle upon Tyne, UK).

### DNA Extraction and Genotyping

Genomic DNA was extracted from buffy-coat and the *TCF7L2-*rs7903146 was genotyped in the whole cohort on a 7900 HT Sequence Detection System (Applied Biosystems, Foster City, CA, USA) using a fluorescent allelic discrimination TaqMan^TM^ assay as previously described^14^.

### Ascertainment of T2D cases

T2D was a pre-specified secondary endpoint of the PREDIMED trial and it was identified by clinical diagnosis and/or use of antidiabetic medication. Information was collected through contact with participants and primary health care physicians, annual follow-up visits, yearly ad-hoc reviews of medical charts and annual consultation of the National Death Index, as it has been described elsewhere^15^. The American Diabetes Association criteria^16^, namely two confirmations of fasting plasma glucose ≥7.0 mmol/L or 2-h plasma glucose ≥11.1 mmol/L, after a 75-g oral glucose load were used to adjudicate incident cases.

### Assessment of covariates and other variables

At baseline and at yearly follow-up visits, a 47-item questionnaire about lifestyle variables, smoking status, medical history and medication use was administered. Physical activity was assessed using a validated Spanish version of the Minnesota Leisure Time Physical Activity Questionnaire^17^. To assess the degree of adherence to Mediterranean diet (MedDiet), a 14-item validated questionnaire was filled in for each participant^18^. Body mass index (BMI) was calculated as weight divided by height squared (kg/m^2^). Participants’ triacylglycerol (TAG), total cholesterol, high-density lipoprotein (HDL) and low-density lipoprotein (LDL) levels were measured using fasting plasma at baseline. Blood glucose and insulin levels were centrally assessed at baseline and at the end of 1-year of follow-up. Insulin resistance was estimated by the HOMA method using the following equation: HOMA-IR was calculated using the following equation:  HOMA-IR = [fasting insulin (μIU/mL) × fasting glucose (mmol/L)]/22.5.

### Statistical analysis

Baseline characteristics of cases and non-cases were described as means and standard deviations (SD) for quantitative variables, and percentages or numbers for categorical variables. We applied a natural logarithmic transformation to approximate a normal distribution of metabolites levels. We also examined product-to-precursor ratios of metabolites, as described in the pathway of purine catabolism, as a metabolite trait (by dividing the raw values and then taking natural logarithmic transformations). Person-time of follow-up was calculated as the interval between the randomization date and date of T2D event, death, or date of the last contact, whichever came first. We used Cox proportional hazard models, with Barlow weights (to account for the over-representation of cases), to estimate hazard ratios (HRs) and their 95% confidence intervals (CIs) for risk of T2D. A crude model and two multivariable-adjusted Cox regression models were fitted as follows: a) multivariable model 1 (MV1), adjusted for age (years), sex (male, female), body mass index (kg/m^2^), intervention group and baseline fasting glucose (mg/dl) (adding a quadratic term to account for the departure from linearity; b) MV2, additionally adjusted for the *TCF7L2*-rs7903146 polymorphism (assuming an additive genetic model), smoking (never, current, former), leisure-time physical activity (metabolic equivalent tasks in minutes/day), baseline dyslipidaemia (yes/no), and hypertension (yes/no). We stratified the models according to recruitment center. Baseline metabolites levels and their ratios were analysed as both continuous variables (1-SD increment in their transformed levels) and using quartiles (using cut-points defined among non-cases). To appraise the linear trend across quartiles, the median metabolites levels and their ratios within each quartile was included in the Cox regression models as a continuous variable. To account for multiple testing, we adjusted p-values of the multivariable adjusted associations between quartiles or 1-SD increment in metabolites levels and T2D risk, using Benjamin-Hochberg false discovery rate (FDR) procedure^19^. A FDR-p-value < 0.05 was considered to be statistically significant. We also examined the associations of 1-year changes in metabolites and their ratios with T2D risk. We used the same models as in the baseline analyses but further adjusted for baseline metabolites levels or their ratios. With respect to metabolites or their ratios, we first calculated the ratio between 1-year and baseline levels and then normalized this ratio with the natural logarithmic transformation. To test the robustness of our findings we conducted one sensitivity analysis on the associations of baseline metabolites or their ratios with risk for T2D by including two variables (HDL and TAG) in the multivariable model 2 that were significantly different between cases and non-cases, leaving 71 incident cases due to the high percentage of missing values (HDL: 41%, TAG: 33%). To examine whether the aforementioned genotypes modified the association between metabolites or relevant ratios and T2D incidence, interactions were tested by including a multiplicative interaction term (metabolite x *TCF7L2*-rs7903146 genotypes) or (ratio x *TCF7L2*-rs7903146 genotypes) and also a *TCF7L2*-rs7903146 genotypes main effect in the MV2. Stratified analyses by *TCF7L2*-rs7903146 genotypes were also carried out assuming an additive genetic model. The associations were adjusted for multiple testing as described above. We also estimated the joint association of plasma metabolites levels and rs7903146 polymorphisms (TT or CC genotypes) with T2D. We considered as the reference group those participants with CC genotype and metabolites levels (lower than the median). Statistical analyses were performed using Stata 13.1 (Stata Corp., College Station, Texas, USA). A two-sided p value less than 0.05 was considered significant.

## Results

### Participants’ characteristics

The median follow-up of the study population was 3.8 years. A total of 251 incident cases and 641 control participants were included in the study. Participants’ characteristics are summarized in Table 1. The mean age of participants at baseline was 66.5 years and the mean BMI was 30.1 (3.5) kg/m^2^. Briefly, a higher proportion of incident cases were men and current smokers compared to non-cases. As compared with non-cases, those participants who developed T2D were also more likely to have a higher prevalence of hypertension and dyslipidaemia in addition to higher BMI, fasting glucose and triacylglycerol levels while lower HDL cholesterol levels (Table 1).

**Table 1:** Baseline characteristics of the study population.

	Total	Cases	Non-cases	p value
n	892	251	641	
Age (years)	66.5 (5.7)	66.4 (5.7)	66.5 (5.7)	0.781
Sex (% Women)	61.2	55.0	63.6	0.017
Body mass index, kg/m^2^	30.1 (3.5)	30.8 (3.3)	29.8 (3.6)	<0.001
Physical activity, METs/d	240.7 (234.6)	249.2 (233.5)	237.4 (235.1)	0.500
**Intervention group, %**				
MedDiet + EVOO	30.6	29.9	30.9	0.425
MedDiet + Nuts	36.3	37.2	33.8	
Control group	33.1	36.3	31.8	
Hypertension, %	91.7	96.0	90.0	0.003
Dyslipidaemia, %	84.3	79.7	86.1	0.018
**Smoking, %**				
Never	59.0	52.6	61.5	0.006
Former	22.4	22.3	22.5	
Current	18.6	25.1	16.0	
Score for adherence to Mediterranean diet^a^	8.5 (2.0)	8.4 (2.0)	8.6 (1.9)	0.186
Fasting blood glucose, mg/dl	103.3 (17.6)	118.6 (18.0)	97.8 (13.8)	<0.001
Total cholesterol, mg/dl	222.2 (39.3)	221.7 (42.3)	222.4 (38.1)	0.846
HDL cholesterol, mg/dL	54.7 (13.0)	52.6 (12.7)	55.7 (13.1)	0.014
LDL cholesterol, mg/dL	139.7 (32.9)	137.0 (31.6)	141.0 (33.3)	0.201
Triacylglycerol, mg/dL	140.1 (85.0)	169.0 (121.0)	128.6 (62.0)	<0.001

Data are mean (SD) or percentage. ^a^This score is based on the 14-item dietary screener. The x^2^ test was used for comparison of categorical variables and Student’s *t*-test was used for comparison of continuous variables. MedDiet, Mediterranean diet; EVOO, Extra-virgin olive oil; MET, metabolic equivalent, HDL, high-density lipoprotein; LDL, low-density lipoprotein.

### Associations of baseline metabolites and relevant ratios (precursor-product) with risk for T2D

Table 2 shows the associations of baseline levels of individual metabolites and relevant ratios with T2D risk. In fully-adjusted model (MV2), allantoin when modelled as quartile, was significantly associated with lower T2D risk [HR in the highest versus lowest quartile was 0.44 (95% CI 0.25–0.80). Regarding ratios of allantoin-to-uric acid and xanthine-to-hypoxanthine, the estimated HR for incident T2D in the highest versus lowest quartile was 0.42 (95% CI 0.22–0.82) and 2.53 (95% CI 1.40–4.58), respectively. These associations remained significant after accounting for multiple comparisons. There were no significant associations between other baseline metabolites or ratios and incident T2D, either they were modelled continuously (per 1 SD) or as quartiles.

**Table 2:** Associations of baseline individual metabolites levels and relevant ratios (precursor-product) with the risk of type 2 diabetes in the PREDIMED study, 2003–2010. Overall group.

Quartiles of plasma metabolite levels									
Metabolite	Q1	Q2	Q3	Q4	p trend	FDR-Adjusted p value (Q4 vs. Q1)	HR per 1 SD increment	p value	FDR-Adjusted p value
**Uric Acid**									
Cases	47	60	58	85					
Crude model	Ref.	1.18 (0.73, 1.91)	1.01 (0.62, 1.64)	1.55 (0.98, 2.45)	0.091		1.16 (0.97, 1.38)	0.098	
MV1	Ref.	1.20 (0.70, 2.05)	0.97 (0.54, 1.74)	1.35 (0.78, 2.35)	0.373		1.09 (0.90, 1.31)	0.363	
MV2	Ref.	1.20 (0.68, 2.11)	0.94 (0.52, 1.71)	1.36 (0.77, 2.40)	0.412	0.637	1.08 (0.88, 1.31)	0.446	0.559
**Allantoin**									
Cases	72	63	72	43					
Crude model	Ref.	0.65 (0.42, 1.02)	0.89 (0.58, 1.37)	0.60 (0.36, 1.01)	0.142		0.97 (0.83, 1.15)	0.787	
MV1	Ref.	0.70 (0.44, 1.13)	0.79 (0.49, 1.28)	0.48 (0.27, 0.85)	0.020		0.89 (0.75, 1.06)	0.193	
MV2	Ref.	0.66 (0.40, 1.08)	0.73 (0.44, 1.22)	0.44 (0.25, 0.80)	0.011	0.045	0.86 (0.73, 1.02)	0.094	0.407
**Xanthine**									
Cases	56	60	64	69					
Crude model	Ref.	1.09 (0.68, 1.76)	1.17 (0.72, 1.90)	1.47 (0.91, 2.37)	0.095		1.21 (1.04, 1.41)	0.015	
MV1	Ref.	1.23 (0.73, 2.07)	1.19 (0.71, 2.00)	1.07 (0.61, 1.88)	0.901		1.08 (0.89, 1.33)	0.408	
MV2	Ref.	1.15 (0.67, 1.99)	1.22 (0.71, 2.09)	1.04 (0.58, 1.87)	0.929	0.886	1.13 (0.92, 1.38)	0.243	0.451
**Hypoxanthine**									
Cases	61	77	55	57					
Crude model	Ref.	1.33 (0.86, 2.06)	0.93 (0.58, 1.48)	1.02 (0.63, 1.66)	0.738		1.00 (0.83, 1.20)	0.977	
MV1	Ref.	1.44 (0.91, 2.29)	0.93 (0.55, 1.57)	0.85 (0.49, 1.47)	0.296		0.93 (0.76, 1.13)	0.472	
MV2	Ref.	1.30 (0.80, 2.11)	0.82 (0.48, 1.42)	0.75 (0.42, 1.33)	0.145	0.637	0.89 (0.72, 1.10)	0.280	0.455
**Inosine**									
Cases	67	48	75	56					
Crude model	Ref.	0.73 (0.45, 1.18)	1.02 (0.65, 1.62)	1.02 (0.62, 1.68)	0.613		1.11 (0.91 1.34)	0.286	
MV1	Ref.	0.73 (0.42, 1.27)	0.90 (0.54, 1.48)	0.92 (0.54, 1.57)	0.995		1.06 (0.86, 1.31)	0.542	
MV2	Ref.	0.75 (0.42, 1.33)	0.98 (0.58, 1.63)	0.89 (0.50, 1.58)	0.939	0.798	1.05 (0.84, 1.31)	0.646	0.699
**Adenosine**									
Cases	42	62	82	62					
Crude model	Ref.	1.06 (0.64, 1.76)	1.35 (0.84, 2.19)	0.90 (0.54, 1.48)	0.774		1.03 (0.88, 1.21)	0.700	
MV1	Ref.	0.98 (0.56, 1.71)	1.34 (0.80, 2.25)	0.72 (0.40, 1.31)	0.374		0.89 (0.72, 1.09)	0.275	
MV2	Ref.	0.88 (0.50, 1.55)	1.21 (0.72, 2.05)	0.64 (0.34, 1.19)	0.224	0.516	0.85 (0.68, 1.06)	0.162	0.451
**Guanosine**									
Cases	44	57	71	77					
Crude model	Ref.	0.92 (0.56, 1.51)	1.04 (0.64, 1.69)	1.15 (0.71, 1.88)	0.461		1.00 (0.80, 1.24)	0.992	
MV1	Ref.	0.93 (0.51, 1.70)	1.09 (0.60, 1.95)	1.22 (0.67, 2.22)	0.412		1.12 (0.88, 1.42)	0.361	
MV2	Ref.	0.95 (0.50, 1.78)	1.06 (0.58, 1.94)	1.34 (0.73, 2.46)	0.281	0.637	1.17 (0.89, 1.54)	0.239	0.451
**Ratio of metabolites**									
**Inosine-to-Adenosine Ratio**									
Cases	54	61	44	58					
Crude model	Ref.	1.04 (0.64, 1.68)	0.79 (0.47, 1.32)	1.04 (0.62, 1.76)	0.821		0.84 (0.68, 1.03)	0.104	
MV1	Ref.	1.13 (0.66, 1.92)	0.95 (0.55, 1.64)	1.15 (0.65, 2.02)	0.761		0.86 (0.68, 1.09)	0.221	
MV2	Ref.	1.17 (0.66, 2.07)	0.93 (0.51, 1.70)	1.20 (0.66, 2.21)	0.712	0.787	0.89 (0.71, 1.13)	0.349	0.504
**Uric Acid-to-Xanthine Ratio**									
Cases	56	59	71	65					
Crude model	Ref.	0.74 (0.47, 1.17)	0.91 (0.57, 1.45)	0.83 (0.52, 1.31)	0.586		0.88 (0.74, 1.05)	0.161	
MV1	Ref.	0.94 (0.53, 1.66)	1.13 (0.67, 1.89)	0.99 (0.58, 1.70)	0.886		0.96 (0.77, 1.19)	0.706	
MV2	Ref.	0.87 (0.49, 1.56)	1.16 (0.68, 1.98)	0.83 (0.47, 1.47)	0.731	0.787	0.92 (0.73, 1.15)	0.473	0.559
**Allantoin-to-Uric Acid Ratio**									
Cases	73	70	69	37					
Crude model	Ref.	0.78 (0.50, 1.20)	0.79 (0.52, 1.22)	0.57 (0.33, 0.98)	0.052		0.91 (0.78, 1.07)	0.283	
MV1	Ref.	0.77 (0.48, 1.25)	0.89 (0.56, 1.42)	0.43 (0.22, 0.82)	0.023		0.85 (0.72, 1.01)	0.074	
MV2	Ref.	0.77 (0.46, 1.27)	0.91 (0.57, 1.45)	0.42 (0.22, 0.82)	0.023	0.047	0.84 (0.71, 0.98)	0.035	0.234
**Xanthine-to-Guanosine Ratio**									
Cases	62	54	77	55					
Crude model	Ref.	0.97 (0.61, 1.57)	1.31 (0.84, 2.06)	1.42 (0.88, 2.30)	0.077		1.22 (1.04, 1.43)	0.013	
MV1	Ref.	0.96 (0.58, 1.60)	1.16 (0.71, 1.91)	0.87 (0.49, 1.55)	0.762		1.02 (0.84, 1.25)	0.803	
MV2	Ref.	0.98 (0.58, 1.66)	1.22 (0.72, 2.06)	0.87 (0.48, 1.58)	0.742	0.798	1.03 (0.84, 1.29)	0.727	0.727
**Xanthine-to-Hypoxanthine Ratio**									
Cases	43	70	57	80					
Crude model	Ref.	1.21 (0.75, 1.96)	1.06 (0.64, 1.76)	1.86 (1.14, 3.02)	0.290		1.21 (1.03, 1.42)	0.017	
MV1	Ref.	1.01 (0.59, 1.72)	1.07 (0.61, 1.91)	1.84 (1.08, 3.15)	0.095		1.14 (0.95, 1.38)	0.157	
MV2	Ref.	1.16 (0.65, 2.07)	1.32 (0.70, 2.47)	2.53 (1.40, 4.58)	0.085	0.026	1.23 (1.01, 1.50)	0.036	0.234
**Hypoxanthine-to-Inosine Ratio**									
Cases	72	57	54	66					
Crude model	Ref.	0.61 (0.39, 0.96)	0.63 (0.39, 1.01)	0.77 (0.48, 1.24)	0.301		0.89 (0.73, 1.08)	0.244	
MV1	Ref.	0.68 (0.42, 1.10)	0.58 (0.33, 1.00)	0.93 (0.56, 1.55)	0.561		0.87 (0.70, 1.08)	0.212	
MV2	Ref.	0.69 (0.41, 1.13)	0.63 (0.35, 1.11)	0.91 (0.53, 1.55)	0.552	0.798	0.86 (0.69, 1.08)	0.201	0.451

Abbreviations: MV, multivariable model; SD, standard deviation. A natural logarithmic transformation was applied to the raw value of individual metabolites. In the case of ratios of metabolites characterised by a precursor-product relationship, their raw values underwent natural logarithmic transformation. Cox regression analysis. MV1: Adjusted for age (years), sex (male, female), body mass index (kg/m^2^), intervention group (MedDiet + EVOO, MedDiet + nuts) and baseline fasting glucose (mg/dl) (centered on the sample mean and adding quadratic term). MV2: additionally adjusted for *TCF7L2*-rs7903146 genotype (assuming an additive genetic model), smoking (never, current, former), leisure-time physical activity (metabolic equivalent tasks in minutes/day), dyslipidemia and hypertension. False discovery rate (FDR) controlling adjustments were conducted by applying the method of Benjamini and Hochberg. MV, multivariable model.

### One year changes in levels of metabolites, relevant ratios (precursor-product) and risk of T2D

Associations between 1-year changes in metabolites levels and relevant ratios across quartiles with the risk of T2D are shown in Supplemental Table 1. In the highest quartile of increase in inosine-to-adenosine and xanthine-to-guanosine ratio a significant inverse association with T2D risk was found, with HR 0.43 (95% CI 0.19–0.95), and HR 0.42 (95% CI 0.20–0.88), respectively. We repeated the analyses using 1-SD increment in 1-year changes of metabolite levels and relevant ratios and found that per 1-SD increase in xanthine-to-guanosine ratio, the risk of T2D was associated with a decrease of 22%, with HR 0.78 (95% CI 0.62–0.98) (Supplemental Table 2). After adjusting for multiple testing, none of these associations remained statistically significant.

### Sensitivity analysis

Supplemental Table 3 shows the associations of baseline levels of individual metabolites and relevant ratios with T2D risk after including HDL cholesterol and TAG in the fully adjusted model. The associations between allantoin and allantoin-to-uric acid ratio with T2D risk remained significant.

### Spearman’s correlation analysis

The correlations between baseline metabolites, relevant ratios and baseline HOMA-IR are presented in Table 3. Several metabolites including uric acid, guanosine, xanthine and adenosine were found to be positively correlated with HOMA-IR (p < 0.05). The ratio of xanthine-to-hypoxanthine was also positively correlated with HOMA-IR (r = 0.09, p = 0.015). On the other hand, inosine-to-adenosine and allantoin-to-uric acid ratios were negatively correlated with HOMA-IR (r = −0.09, p = 0.013; r = −0.07, p = 0.037). Correlation analysis between 1-year changes in levels of metabolites, relevant ratios and 1-year changes in HOMA-IR (increase in HOMA-IR levels) revealed significant positive correlations for uric acid (r = 0.14, p = 0.001), allantoin (r = 0.09, p = 0.026), guanosine (r = 0.13, p = 0.002) and xanthine-to-hypoxanthine ratio (r = 0.10, p = 0.024).

**Table 3:** Spearman’s correlation analysis between baseline metabolites levels, relevant ratios and baseline HOMA-IR.

	Spearman r	p value
Uric Acid	0.22	<0.001
Allantoin	0.01	0.682
Xanthine	0.13	0.001
Hypoxanthine	0.02	0.644
Inosine	0.01	0.719
Adenosine	0.11	0.003
Guanosine	0.16	<0.001
Inosine-to-Adenosine	−0.09	0.013
Uric Acid-to-Xanthine	0.04	0.214
Allantoin-to-Uric Acid	−0.07	0.037
Xanthine-to-Guanosine	0.04	0.266
Xanthine-to-Hypoxanthine	0.09	0.015
Hypoxanthine-to-Inosine	0.01	0.821

### Interaction between the *TCF7L2*-rs7903146 polymorphism and metabolites on T2D risk

**Figure 1:**
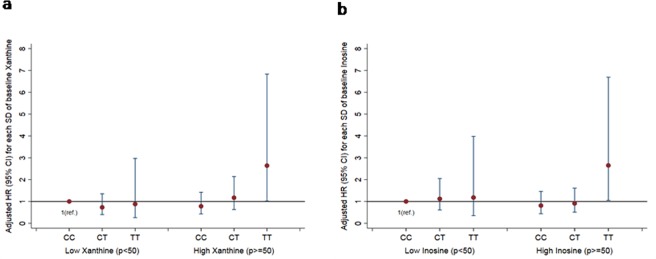
(**a**) Joint association of plasma xanthine levels and *TCF7L2*-rs7903146 genotypes in relation to type 2 diabetes risk. The number 50 corresponds to 50th percentile; median. (**b**) Joint association of plasma inosine levels and *TCF7L2*-rs7903146 genotypes in relation to type 2 diabetes risk. The number 50 corresponds to 50th percentile; median.

As expected, TT individuals had a higher risk for T2D compared with CC homozygotes, with HR 2.03 (95% CI 1.12–3.70), p = 0.020, after controlling for several potential confounding factors. No excess risk was conferred by the heterozygous genotype [CT *vs*. CC: HR 1.19 (95% CI 0.79–1.79), p = 0.387]. We found statistically significant interactions between metabolites levels, a relevant ratio and the *TCF7L2*-rs7903146 polymorphism in determining T2D risk in an additive model (Table 4). We found that per 1-SD increase in plasma xanthine and inosine levels, the risk of T2D significantly increased in TT individuals, with HR 2.34 (95% CI 1.23–4.45), and HR 3.09 (95% CI 1.23–7.73), respectively. On the other hand, per 1-SD increase in hypoxanthine-to-inosine ratio, a 74% lower risk of T2D was found in TT subjects [HR 0.26 (95% CI 0.10–0.70)]. After adjustment for multiple comparisons the associations between these metabolites, the ratio and T2D risk remained statistically significant. When the joint effects were examined, individuals with TT genotype and plasma xanthine levels above/equal to the median value had significantly higher risk of T2D [HR 2.64 (95% CI 1.02–6.83)] than those with CC genotype and xanthine levels below the median (reference) (Fig. 1a). Similarly, higher risk was found in TT individuals when levels of inosine were higher or equal to the median [HR 2.65 (95% CI 1.05–6.69)] (Fig. 1b). The levels of the aforementioned metabolites did not significantly differ across the genotypes in the additive model.

**Table 4:** Associations of baseline individual metabolites levels and relevant ratios (precursor-product) with the risk of type 2 diabetes by transcription factor-7-like 2 (*TCF7L2*) rs7903146 genotype (additive model: TT & CT & CC).

	CC	CT	TT	FDR-Adjusted p value*	p interaction
n	382	383	104		
Cases, n	106	103	35		
Per SD of change	HR (95% CI)	HR (95% CI)	HR (95% CI)		
**Metabolite**					
Uric Acid	1.08 (0.82, 1.42)	0.97 (0.71, 1.32)	1.35 (0.48, 3.84)	0.668	0.540
Allantoin	0.92 (0.73, 1.15)	0.92 (0.63, 1.34)	0.74 (0.58, 0.93)	0.026	0.581
Xanthine	1.01 (0.73, 1.40)	1.37 (0.99, 1.89)	2.34 (1.23, 4.45)	0.026	0.009
Hypoxanthine	0.95 (0.69, 1.31)	0.83 (0.59, 1.17)	1.23 (0.29, 5.27)	0.842	0.238
Inosine	0.89 (0.61, 1.28)	1.05 (0.78, 1.41)	3.09 (1.23, 7.73)	0.029	0.009
Adenosine	0.95 (0.71, 1.27)	0.70 (0.47, 1.03)	0.32 (0.04, 2.62)	0.371	0.190
Guanosine	0.97 (0.78, 1.20)	1.75 (1.07, 2.87)	1.02 (0.26, 3.92)	0.978	<0.001
**Ratio of metabolites**					
Inosine-to-Adenosine Ratio	0.99 (0.71, 1.39)	0.87 (0.58, 1.30)	0.33 (0.14, 0.78)	0.026	0.083
Uric Acid-to-Xanthine Ratio	1.04 (0.70, 1.55)	0.74 (0.54, 1.02)	0.46 (0.19, 1.14)	0.135	0.032
Allantoin-to-Uric Acid Ratio	0.87 (0.69, 1.11)	0.94 (0.64, 1.38)	0.74 (0.57, 0.95)	0.032	0.520
Xanthine-to-Guanosine Ratio	1.04 (0.76, 1.42)	1.03 (0.69, 1.52)	2.91 (1.42, 5.97)	0.026	0.068
Xanthine-to-Hypoxanthine Ratio	1.05 (0.76, 1.46)	1.55 (1.12, 2.14)	2.84 (1.26, 6.38)	0.026	0.372
Hypoxanthine-to-Inosine Ratio	1.15 (0.78, 1.71)	0.83 (0.62, 1.11)	0.26 (0.10, 0.70)	0.026	0.004

Product (*TCF7L2* × metabolite or ratio) interaction term was included into the model. The PREDIMED study, 2003–2010.

Abbreviations: SD, standard deviation.

^a^A natural logarithmic transformation was applied to the raw values individual metabolites. In the case of ratios of metabolites characterised by a precursor-product relationship, their raw values underwent natural logarithmic transformation. Cox regression analysis.

^b^Stratified by recruitment center; ^c^Adjusted for baseline fasting glucose (mg/dl) (centered on the sample mean and adding quadratic term), age (years), sex (male, female), intervention group (MedDiet + EVOO, MedDiet + nuts), body mass index (kg/m^2^), smoking (never, current, former), leisure-time physical activity (metabolic equivalent tasks in minutes/day), dyslipidemia and hypertension. False discovery rate (FDR) controlling adjustments were conducted by applying the method of Benjamini and Hochberg.

*Homozygous carriers of the *TCF7L2*-rs7903146 T-allele.

## Discussion

In the present case-cohort design study within the PREDIMED trial, after adjusting for recognized T2D risk-factors and multiple testing, we found inverse and positive associations between high baseline levels of allantoin, including allantoin-to-uric acid ratio and high xanthine-to-hypoxanthine ratio with T2D risk, respectively. We also found that elevated plasma xanthine and inosine levels were associated with a higher T2D risk in individuals with TT alleles on rs7903146. This is the first time, as far as we know^10,11^, that this genetic variant of the *TCF7L2* gene, which mediates high susceptibility to T2D, is reported to interact with purine-catabolism metabolites.

**Figure 2:**
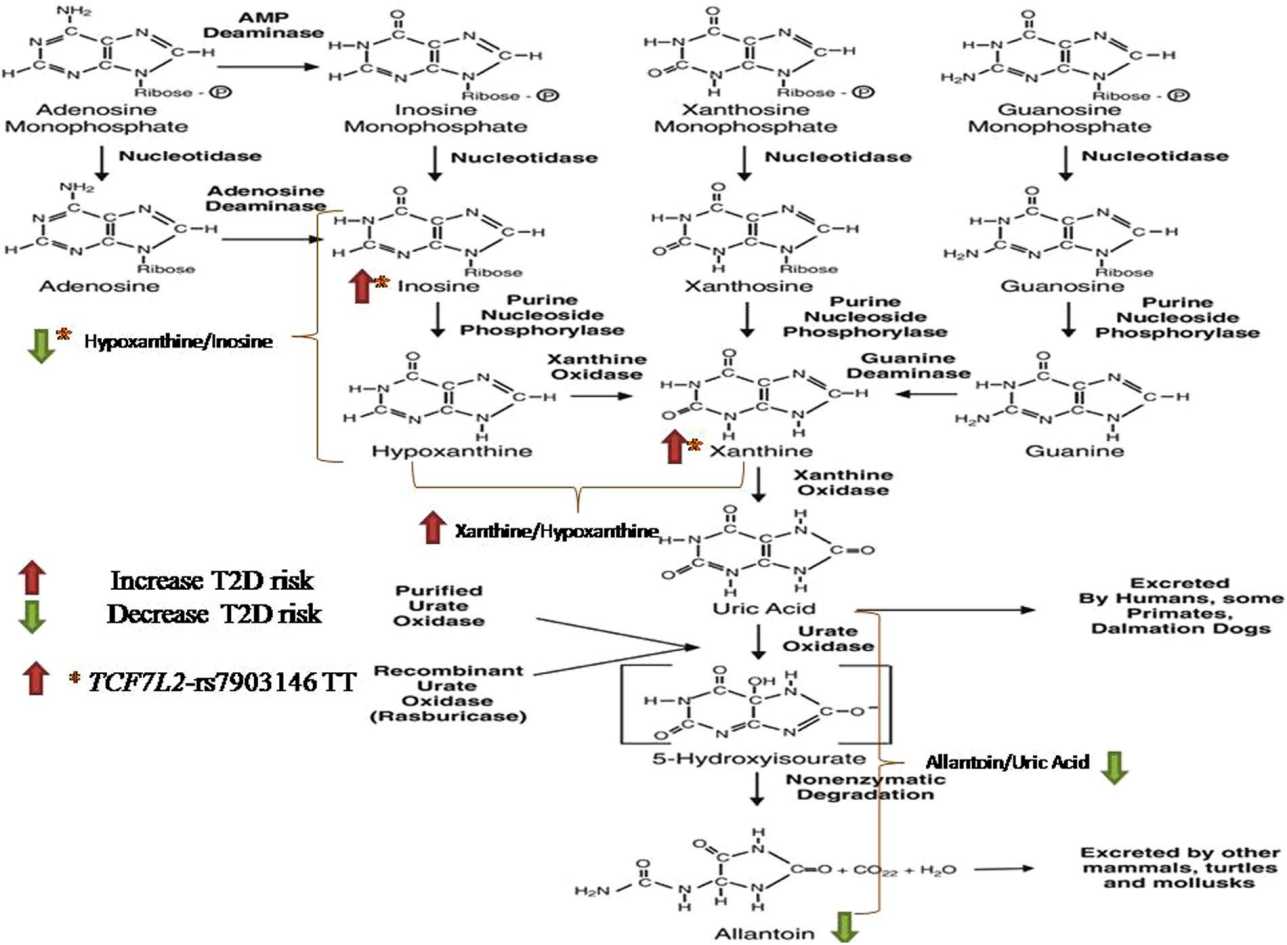
Purine Catabolism Pathway. Reproduced and modified from^41^.

Allantoin is produced from the non-enzymatic oxidation of uric acid (Fig. 2) in humans and is considered to be a specific biomarker of oxidative stress^3,20^. We found that higher allantoin levels were associated with lower risk of T2D incidence as was the allantoin-to-uric acid ratio. This ratio was also negatively correlated with HOMA-IR at baseline. Our results are in accordance with the literature. When administered through injection, allantoin decreased blood glucose levels and increased blood insulin in a dose-dependent manner, in normal rats^21^. Allantoin has also been found to lower plasma glucose in diabetic rats by activating imidazoline-receptors I2 or I3^21^, which improve insulin action. Furthermore, in diabetic rats, allantoin offered through diet had antidiabetic effects by modulating antioxidant activities and lipid profiles, promoting glucagon-like peptide-1 (GLP-1) release, thereby facilitating β-cells to maintain insulin and glucose levels^22^. Finally, in a cohort study, allantoin measured in urine of humans was also inversely associated with the risk of T2D^20^.

Uric acid is the end product of purine metabolism in humans. In our study, plasma uric acid was not associated with future risk of T2D, but correlated positively both with HOMA-IR at baseline and at 1-year follow-up. The causal association between serum uric acid levels and T2D remains controversial^23^. Uric acid is generally considered to be a feature of hyperinsulinemia or/and insulin resistance^23^; however, in patients with diabetes, its level may be low, due to increased urate clearance that can be associated with glycosuria, increased sodium (Na) secretion, and overall decreased metabolic control^24^.

Uric acid can be produced from xanthine via the enzymatic action of XO, which also participates in the conversion of hypoxanthine-to-xanthine (Fig. 2). In our study, baseline xanthine levels and xanthine-to-hypoxanthine ratio were correlated with higher HOMA-IR levels. In addition, we found that this ratio was associated with increased risk of T2D. An increase in this ratio may reflect higher XO activity, which is involved in free radical production^25^ and has been found, increased in T2D patients^5^. Whether xanthine production from hypoxanthine, plays a role in T2D development due to increased XO activity or due to xanthine production in parallel with the consequent decrease in hypoxanthine, requires further investigation.

In the present study, guanosine was also positively correlated to HOMA-IR. Cyclic Guanosine monophosphate (cGMP) is a second messenger that mediates incretin effects; potentiates glucose-stimulated insulin secretion; promotes proper beta-cells differentiation, and prevents beta-cells apoptosis, cooperating with biotin^26^. In addition, cGMP is involved in various signal-transduction pathways, mediating messages of insulin itself^27^. The interactions between the extracellular and intracellular guanosine metabolites, or/and the possible modulations of the latter in T2D, remain an open issue.

Inosine, a precursor of xanthine that was not associated to diabetes incidence in the overall population of our study, has notable anti-inflammatory effects, which may be mediated, at least in part, by activation of the adenosine A2a receptor^28^. Both, adenosine and inosine have been suggested to play a protective role against diabetes development^28^. We observed that inosine-to-adenosine ratio was negatively correlated with baseline HOMA-IR. A working hypothesis is that the production of inosine is more favourable in improving glucose homeostasis, as compared to adenosine.

The *TCF7L2*-rs7903146 polymorphism is one of the strongest and most widely replicated locus associated with T2D^29^, with the homozygous individuals (TT) being those who present a higher prevalence and fasting glucose; the mechanism has yet to be determined^14^, although various hypotheses, affecting regulation of the Wnt signalling-pathway^30^ have been proposed. The key effector of Wnt pathway, the bipartite transcription factor β-cat/TCF, is formed by free β-catenin (β-cat) and a TCF protein, including *TCF7L2*^31^. Wnt signalling and *TCF7L2* appear to exercise a very complex effect in metabolic homeostasis, affecting not only the pancreatic islets, but also other organs (liver gluconeogenesis), and crosstalk with stress, aging as well as tumorigenesis signalling pathways/cascades^31^. Several signalling components of the Wnt signal transduction pathway have been identified but a clear understanding of the Wnt signalling’s diverse function, integration and specificity is lacking. On the other hand, there is strong evidence for a direct association between dysregulated Wnt signalling and chronic diseases^32^. In due course, choosing the rs7903146-allele, was an innovative approach in our study.

In this study, using a case-cohort design, we confirmed the association of the *TCF7L2*-rs7903146 TT genotype, with the risk of T2D in PREDIMED study participants^14^. Changes in the blood of TT homozygotes reported until now mainly concerned increased levels of plasma glucose in response to a meal challenge of proinsulin and elevated glucose-dependent insulinotropic peptide secretion^33^; reduced secretion of insulin/glucagon, and reduced insulinotropic effect of incretin hormones^34^; altered postprandial triglyceride response, mainly influencing VLDL and HDL subclasses^35^, as well as non-significant increase of plasma sphingomyelins, phosphatidylcholines and lyso-phosphatidylcholines species^36^. To our knowledge, this is the first study to examine the interaction between plasma purine-catabolism metabolites levels and the *TCF7L2*-rs7903146 genetic variation, focusing on T2D risk.

We observed that xanthine and inosine were associated with increased T2D risk only in individuals with TT alleles on rs7903146. Moreover, the combination of TT genotype and high plasma xanthine and inosine levels was associated with a higher risk of T2D than the combined CC genotype and their low levels, which confirmed that both the rs7903146 T allele and increased plasma xanthine and inosine are associated with T2D. The protective role of inosine is well-documented^37^, while xanthine may simply increase due to increased XO activity in the blood^38^. A working hypothesis is that xanthine and inosine play a compensatory role, competing or cooperating; since in both, when their levels were low, no significant association was found between TT and T2D, as compared to their high levels (see joint analysis). Among purine-catabolism related ratios, only hypoxanthine-to-inosine ratio was inversely associated with T2D risk in individuals with TT alleles on rs7903146 with a significant test of interaction. Whether hypoxanthine production from inosine confers higher protection from T2D in individuals carrying these polymorphisms, by inhibiting the activation of poly(ADP-ribose) polymerase^39^ and thus increasing *TCF7L2*-mediated GLP-1 production and activity^40^, needs to be further explored.

The results of the present study should be interpreted in the context of its limitations and strengths. First, participants were elderly Mediterranean individuals at high cardiovascular risk and this may limit the generalizability of the findings to other age-groups or populations. Second, even though we adjusted for several potential confounders, residual confounding may exist. Regarding strengths, the prospective evaluation of the association between metabolites levels and well-documented incident T2D, in the frame of a case-cohort design, minimizes biases that can affect case-control studies.

In conclusion, our prospective study documented, for the first time, an inverse and a positive association between high plasma allantoin levels, including allantoin-to-uric acid ratio and high xanthine-to-hypoxanthine ratio with incident T2D risk, respectively, in an elderly population at high cardiovascular risk, independently of the *TCF7L2*-rs7903146 polymorphism. Elevated plasma levels of xanthine and inosine appeared to be associated with higher T2D risk only in TT individuals. These results must be interpreted cautiously and need to be replicated in other populations. The potential mechanisms linking the aforementioned purine metabolites and T2D risk must be also further investigated.

## Supplementary information

SUPPLEMENTARY INFORMATION
