# Supplementary material for: Metabolites related to purine catabolism and risk of type 2 diabetes incidence; modifying effects of the TCF7L2-rs7903146 polymorphism
Source: Sci Rep. 2019 Feb 27;9:2892. doi: 10.1038/s41598-019-39441-6 (PMC6393542; doi:10.1038/s41598-019-39441-6)
Supplement: Supplementary file 1 — SUPPLEMENTARY INFORMATION [file 41598_2019_39441_MOESM1_ESM.docx]

**SUPPLEMENTARY INFORMATION**

**Full title:**

Metabolites related to purine catabolism and risk of type 2 diabetes incidence; modifying effects of the *TCF7L2*-rs7903146 polymorphism

**Authors:**

Christopher Papandreou, Jun Li, Liming Liang, Mònica Bulló, Yan Zheng, Miguel Ruiz-Canela, Edward Yu, Marta Guasch-Ferré, Cristina Razquin, Clary Clish, Dolores Corella, Ramon Estruch, Emilio Ros, Montserrat Fitó, Fernando Arós, Lluís Serra-Majem, Nuria Rosique, Miguel A Martínez-González, Frank B Hu, Jordi Salas-Salvadó.

**Supplemental Figure 1.** Flow-chart of study participants.

Cohort: 3,541 participantswithout T2D at baseline in the PREDIMED study

(273 incident cases)

22 incident cases without available plasma samples

random 20% subcohort (N=694*)

+ all incident cases

**N=892 participants**

Non-cases: 641

+

Cases: 251

Participants with metabolites measured at baseline

206 without plasma samples available or with incident T2D within the 1st year of follow-up *

**N= 686 participants**

Non-cases: 505

+

Cases: 181

Participants with metabolites measured at year 1

*53 incident cases included in the subcohort

# Supplemental Table 1. Associations of 1-year changes in individual metabolites levels and relevant ratios (precursor-product) with the risk of type 2 diabetes in the PREDIMED study, 2003-2010. Overall group.

|  |  | **Quartiles of plasma metabolite levels** | | | |  |
| --- | --- | --- | --- | --- | --- | --- |
| **Metabolite** | Q1 | Q2 | Q3 | Q4 | p trend | FDR-Adjusted p value (Q4 vs. Q1) |
| **Uric Acid** |  |  |  |  |  |  |
| Cases | 37 | 45 | 53 | 45 |  |  |
| MV | Ref. | 1.34 (0.69, 2.59) | 1.40 (0.73, 2.67) | 1.32 (0.65, 2.67) | 0.437 | 0.719 |
| **Allantoin** |  |  |  |  |  |  |
| Cases | 54 | 35 | 54 | 36 |  |  |
| MV | Ref. | 1.15 (0.63, 2.09) | 1.06 (0.57, 1.96) | 0.93 (0.46, 1.86) | 0.838 | 0.903 |
| **Xanthine** |  |  |  |  |  |  |
| Cases | 50 | 46 | 41 | 42 |  |  |
| MV | Ref. | 0.92 (0.50, 1.68) | 0.87 (0.46, 1.67) | 0.74 (0.38, 1.47) | 0.388 | 0.719 |
| **Hypoxanthine** |  |  |  |  |  |  |
| Cases | 36 | 56 | 44 | 43 |  |  |
| MV | Ref. | 1.47 (0.77, 2.82) | 1.27 (0.64, 2.53) | 1.10 (0.49, 2.46) | 0.985 | 0.903 |
| **Inosine** |  |  |  |  |  |  |
| Cases | 53 | 41 | 41 | 46 |  |  |
| MV | Ref. | 0.54 (0.29, 0.99) | 0.52 (0.27, 1.00) | 0.65 (0.32, 1.361) | 0.304 | 0.719 |
| **Adenosine** |  |  |  |  |  |  |
| Cases | 42 | 39 | 55 | 42 |  |  |
| MV | Ref. | 1.22 (0.62, 2.37) | 1.37 (0.70, 2.65) | 1.31 (0.66, 2.60) | 0.453 | 0.719 |
| **Guanosine** |  |  |  |  |  |  |
| Cases | 50 | 34 | 42 | 50 |  |  |
| MV | Ref. | 0.73 (0.40, 1.34) | 0.72 (0.40, 1.28) | 0.84 (0.44, 1.61) | 0.519 | 0.868 |
| **Ratio of metabolites (precursor-product)** |  |  |  |  |  |  |
| **Inosine-to-Adenosine Ratio** |  |  |  |  |  |  |
| Cases | 57 | 43 | 38 | 40 |  |  |
| MV | Ref. | 0.49 (0.28, 0.87) | 0.72 (0.41, 1.25) | 0.43 (0.19, 0.95) | 0.038 | 0.247 |
| **Uric Acid-to-Xanthine Ratio** |  |  |  |  |  |  |
| Cases | 43 | 45 | 31 | 60 |  |  |
| MV | Ref. | 1.49 (0.81, 2.74) | 0.76 (0.37, 1.56) | 1.33 (0.72, 2.48) | 0.682 | 0.719 |
| **Allantoin-to-Uric Acid Ratio** |  |  |  |  |  |  |
| Cases | 60 | 39 | 49 | 33 |  |  |
| MV | Ref. | 0.88 (0.49, 1.58) | 1.25 (0.69, 2.29) | 0.60 (0.30, 1.20) | 0.240 | 0.663 |
| **Xanthine-to-Guanosine Ratio** |  |  |  |  |  |  |
| Cases | 58 | 46 | 42 | 34 |  |  |
| MV | Ref. | 0.90 (0.51, 1.60) | 0.86 (0.47, 1.56) | 0.42 (0.20, 0.88) | 0.021 | 0.247 |
| **Xanthine-to-Hypoxanthine Ratio** |  |  |  |  |  |  |
| Cases | 45 | 48 | 41 | 45 |  |  |
| MV | Ref. | 1.19 (0.67, 2.11) | 0.79 (0.41, 1.52) | 1.08 (0.53, 2.20) | 0.970 | 0.903 |
| **Hypoxanthine-to-Inosine Ratio** |  |  |  |  |  |  |
| Cases | 45 | 43 | 42 | 51 |  |  |
| MV | Ref. | 1.04 (0.54, 1.97) | 0.72 (0.37, 1.39) | 0.98 (0.50, 1.90) | 0.713 | 0.950 |

Abbreviations: MV, multivariable model. A natural logarithmic transformation was applied to the raw values of the ratio between 1-year and baseline levels in individual metabolites. In the case of ratios of metabolites characterised by a precursor-product relationship, their raw values underwent natural logarithmic transformation. MV: Adjusted for baseline levels of metabolites, age (years), sex (male, female), intervention group (MedDiet+EVOO, MedDiet+nuts), body mass index (kg/m^2^), baseline fasting glucose (mg/dl) (centered on the sample mean and adding quadratic term), *TCF7L2*-rs7903146 genotype (assuming an additive genetic model), smoking (never, current, former), leisure-time physical activity (metabolic equivalent tasks in minutes/day), dyslipidemia and hypertension. False discovery rate (FDR) controlling adjustments were conducted by applying the method of Benjamini and Hochberg.

**Supplemental Table 2.** Associations of 1-year changes (per standard deviation) in individual metabolites levels and relevant ratios (precursor-product) with the risk of type 2 diabetes. The PREDIMED study, 2003-2010.

|  | **Overall group** | **Unadjusted p value** | **FDR-Adjusted p value** |
| --- | --- | --- | --- |
| **Subcohort, n** | 546 |  |  |
| **Cases, n** | 181 |  |  |
| **Per SD of change** | HR (95%CI) |  |  |
| **Metabolite** |  |  |  |
| **Uric Acid** | 1.02 (0.80, 1.31) | 0.855 | 0.855 |
| **Allantoin** | 1.07 (0.87, 1.32) | 0.498 | 0.803 |
| **Xanthine** | 0.93 (0.74, 1.46) | 0.549 | 0.803 |
| **Hypoxanthine** | 0.92 (0.68, 1.26) | 0.618 | 0.803 |
| **Inosine** | 0.87 (0.66, 1.14) | 0.307 | 0.803 |
| **Adenosine** | 1.12 (0.90, 1.39) | 0.319 | 0.803 |
| **Guanosine** | 1.21 (0.90, 1.64) | 0.199 | 0.803 |
| **Ratio of metabolites** |  |  |  |
| **Inosine-to-Adenosine Ratio** | 0.85 (0.68, 1.07) | 0.162 | 0.803 |
| **Uric Acid-to-Xanthine Ratio** | 1.06 (0.85, 1.33) | 0.591 | 0.803 |
| **Allantoin-to-Uric Acid Ratio** | 1.05 (0.81, 1.35) | 0.722 | 0.853 |
| **Xanthine-to-Guanosine Ratio** | 0.78 (0.62, 0.98) | 0.037 | 0.481 |
| **Xanthine-to-Hypoxanthine Ratio** | 1.02 (0.79, 1.32) | 0.845 | 0.855 |
| **Hypoxanthine-to-Inosine Ratio** | 1.10 (0.85, 1.42) | 0.475 | 0.803 |

Abbreviations: MedDiet, Mediterranean Diet; SD, standard deviation.

^a^ A natural logarithmic transformation was applied to the raw values of the ratio between 1-year and baseline levels in individual metabolites. In the case of ratios of metabolites characterised by a precursor-product relationship, their raw values underwent natural logarithmic transformation.

^b^ Stratified by recruitment center; ^c^ Adjusted for baseline levels of metabolites, baseline fasting glucose (mg/dl) (centered on the sample mean and adding quadratic term), age (years), sex (male, female), intervention group (MedDiet+EVOO, MedDiet+nuts) (except in analyses by intervention group), body mass index (kg/m^2^), *TCF7L2*-rs7903146 genotype (assuming an additive genetic model), smoking (never, current, former), leisure-time physical activity (metabolic equivalent tasks in minutes/day), dyslipidemia and hypertension. False discovery rate (FDR) controlling adjustments were conducted by applying the method of Benjamini and Hochberg.

**Supplemental Table 3.** Associations of baseline individual metabolites levels and relevant ratios (precursor-product) with the risk of type 2 diabetes (71 incident cases remained after adjustment for HDL cholesterol and triacylglycerol) in the PREDIMED study, 2003-2010. Overall group.

|  |  | |  |  | | | | | | | | | | |
| --- | --- | --- | --- | --- | --- | --- | --- | --- | --- | --- | --- | --- | --- | --- |
|  | |  | | |  | | **Quartiles of plasma metabolite levels** | | | | |  | |  |
| **Metabolite** | | Q1 | | | Q2 | | | Q3 | Q4 | p trend | FDR-Adjusted p value (Q4 vs. Q1) | |  |  |
| **Uric Acid** | |  | | |  | | |  |  |  |  | |  |  |
| MV | | Ref. | | | 2.64 (0.87, 8.03) | | | 0.69 (0.18, 2.57) | 1.52 (0.47, 4.91) | 0.741 | 0.691 | |  |  |
| **Allantoin** | |  | | |  | | |  |  |  |  | |  |  |
| MV | | Ref. | | | 0.26 (0.11, 0.61) | | | 0.54 (0.22, 1.31) | 0.17 (0.05, 0.55) | 0.006 | 0.039 | |  |  |
| **Xanthine** | |  | | |  | | |  |  |  |  | |  |  |
| MV | | Ref. | | | 0.52 (0.17, 1.58) | | | 0.61 (0.19, 1.88) | 2.10 (0.61, 7.15) | 0.222 | 0.511 | |  |  |
| **Hypoxanthine** | |  | | |  | | |  |  |  |  | |  |  |
| MV | | Ref. | | | 1.25 (0.59, 2.65) | | | 0.88 (0.33, 2.28) | 0.70 (0.20, 2.45) | 0.531 | 0.756 | |  |  |
| **Inosine** | |  | | |  | | |  |  |  |  | |  |  |
| MV | | Ref. | | | 1.36 (0.56, 3.30) | | | 0.92 (0.33, 2.55) | 0.91 (0.27, 3.06) | 0.771 | 0.913 | |  |  |
| **Adenosine** | |  | | |  | | |  |  |  |  | |  |  |
| MV | | Ref. | | | 1.25 (0.38, 4.07) | | | 1.46 (0.46, 4.64) | 1.15 (0.35, 3.76) | 0.809 | 0.913 | |  |  |
| **Guanosine** | |  | | |  | | |  |  |  |  | |  |  |
| MV | | Ref. | | | 0.38 (0.14, 1.04) | | | 0.51 (0.20, 1.29) | 0.43 (0.15, 1.21) | 0.190 | 0.397 | |  |  |
| **Ratio of metabolites** | |  | | |  | | |  |  |  |  | |  |  |
| **Inosine-to-Adenosine Ratio** | |  | | |  | | |  |  |  |  | |  |  |
| MV | | Ref. | | | 0.87 (0.29, 2.59) | | | 1.18 (0.37, 3.83) | 1.07 (0.29, 3.97) | 0.856 | 0.913 | |  |  |
| **Uric Acid-to-Xanthine Ratio** | |  | | |  | | |  |  |  |  | |  |  |
| MV | | Ref. | | | 0.85 (0.31, 2.37) | | | 0.31 (0.09, 1.02) | 0.44 (0.14, 1.35) | 0.081 | 0.397 | |  |  |
| **Allantoin-to-Uric Acid Ratio** | |  | | |  | | |  |  |  |  | |  |  |
| MV | | Ref. | | | 0.57 (0.23, 1.45) | | | 0.62 (0.27, 1.46) | 0.18 (0.05, 0.61) | 0.007 | 0.039 | |  |  |
| **Xanthine-to-Guanosine Ratio** | |  | | |  | | |  |  |  |  | |  |  |
| MV | | Ref. | | | 0.73 (0.20, 2.59) | | | 2.69 (1.06, 6.81) | 1.55 (0.46, 5.26) | 0.149 | 0.691 | |  |  |
| **Xanthine-to-Hypoxanthine Ratio** | |  | | |  | | |  |  |  |  | |  |  |
| MV | | Ref. | | | 0.96 (0.26, 3.47) | | | 1.44 (0.31, 6.67) | 2.95 (0.71, 12.22) | 0.106 | 0.397 | |  |  |
| **Hypoxanthine-to-Inosine Ratio** | |  | | |  | | |  |  |  |  | |  |  |
| MV | | Ref. | | | 0.35 (0.13, 0.95) | | | 0.66 (0.25, 1.71) | 0.63 (0.21, 1.86) | 0.586 | 0.691 | |  |  |
|  | |  | | | |  | | | | | |  | |  |

Abbreviations: MV, multivariable model; SD, standard deviation. A natural logarithmic transformation was applied to the raw value of individual metabolites. In the case of ratios of metabolites characterised by a precursor-product relationship, their raw values underwent natural logarithmic transformation. Cox regression analysis. MV: Adjusted for age (years), sex (male, female), body mass index (kg/m^2^), intervention group (MedDiet+EVOO, MedDiet+nuts), baseline fasting glucose (mg/dl) (centered on the sample mean and adding quadratic term), *TCF7L2*-rs7903146 genotype (assuming an additive genetic model), smoking (never, current, former), leisure-time physical activity (metabolic equivalent tasks in minutes/day), dyslipidemia, hypertension, HDL cholesterol and triacylglycerol. False discovery rate (FDR) controlling adjustments were conducted by applying the method of Benjamini and Hochberg. MV, multivariable model.

**Supplemental Table 4.** Spearman's correlation analysis between 1-year changes in individual metabolites levels, relevant ratios (precursor-product) and 1-year changes in HOMA-IR.

|  | **Spearman r** | **p value** |
| --- | --- | --- |
| Uric Acid | 0.14 | 0.001 |
| Allantoin | 0.09 | 0.026 |
| Xanthine | 0.03 | 0.393 |
| Hypoxanthine | -0.06 | 0.159 |
| Inosine | -0.05 | 0.254 |
| Adenosine | -0.00 | 0.980 |
| Guanosine | 0.13 | 0.002 |
| Inosine-to-Adenosine | -0.01 | 0.661 |
| Uric Acid-to-Xanthine | 0.07 | 0.088 |
| Allantoin-to-Uric Acid | 0.01 | 0.703 |
| Xanthine-to-Guanosine | -0.08 | 0.064 |
| Xanthine-to-Hypoxanthine | 0.10 | 0.024 |
| Hypoxanthine-to-Inosine | 0.01 | 0.693 |
